# Ante-mortem cognitive trajectories associated with Aβ and tau biomarker profiles in older adults with cerebrovascular disease: a longitudinal cohort study

**DOI:** 10.1186/s13195-025-01776-w

**Published:** 2025-07-18

**Authors:** Emily Rosenich, Yen Ying Lim, on behalf of the National Alzheimer’s Coordinating Center

**Affiliations:** Turner Institute for Brain and Mental Health, School of Psychological Sciences, Monash University, Melbourne, VIC Australia

**Keywords:** Cognition, Beta-amyloid, Tau, Cerebrovascular disease

## Abstract

**Background:**

Beta-amyloid (Aβ) plaques and tau tangles are pathological hallmarks of Alzheimer’s disease (AD); however, autopsy studies reveal that most older adults also present with cerebrovascular disease (CVD) markers. It remains unclear how Aβ and tau relate to cognition in the context of concurrent CVD. In initially cognitively unimpaired older adults with CVD, this study aimed to determine *ante-mortem* cognitive trajectories associated with elevated Aβ and/or tau at autopsy.

**Methods:**

Participants aged 65–95 classified as cognitively unimpaired at baseline from the National Alzheimer’s Coordinating Center database, with ≥ 1 follow-up between 2005 and 2015, and available autopsy/*APOE* data were included in this cohort study (*N* = 863). All participants had at least one of six CVD markers at autopsy. Participants were classified into four groups (A − T−, A + T−, A − T+, A + T+) based on semiquantitative Consortium to Establish a Registry for Alzheimer’s Disease neuritic plaque staging and Braak staging. Linear mixed models assessed rate of change in Preclinical Alzheimer’s Cognitive Composite scores, episodic memory, and executive function.

**Results:**

A + T + adults demonstrated significantly faster cognitive decline on all outcomes in the ~ 10 years preceding death compared to A − T− adults (d = 0.34–0.46). Similarly, when compared to A + T − adults, A + T + adults showed significantly faster decline on all outcomes (d = 0.19–0.37). At the last visit prior to death, a greater proportion of A + T + adults (36%) received a dementia diagnosis compared to A − T+ (15%; OR = 6.00), A + T− (14%; OR = 8.00) and A − T− adults (12%; OR = 6.86), *p* <.001. When analyses were restricted to exclude dementia diagnoses, significantly faster decline on all outcomes (*p*’s < 0.001, d = 0.29–0.37) was similarly observed in A + T + adults compared to A − T− adults.

**Conclusions:**

In older adults with concurrent CVD, A + T + at autopsy was associated with greater cognitive decline over 10 years preceding death compared to A − T− older adults. Faster cognitive decline in A + T + in the context of low final visit dementia diagnoses may suggest that *post-mortem* A + T + is associated with a steep trajectory of cognitive decline *ante-mortem*, but that dementia progression is not inevitable.

**Supplementary Information:**

The online version contains supplementary material available at 10.1186/s13195-025-01776-w.

## Background

Alzheimer’s disease (AD) is characterized biologically by the presence of beta-amyloid (Aβ) plaques and neurofibrillary tau tangles [1]. Prospective clinicopathological studies have shown that in cognitively unimpaired (CU) individuals, biomarker abnormalities consistent with AD pathophysiologic change (e.g., A + T+) are detectable early, and associated with hippocampal volume loss, cognitive decline, and an increased risk of progression to mild cognitive impairment (MCI) and dementia [1–6]. However, autopsy studies reveal that up to 80% of clinically diagnosed AD dementia cases also present with other pathologies, particularly markers of cerebrovascular disease (CVD), such as small vessel disease, micro- and macro-infarcts, and cerebral amyloid angiopathy [7, 8]. While Aβ and tau account for a considerable proportion of AD dementia cases, CVD can also exacerbate disease progression [9]. For example, one study of 393 CU adults enrolled in the Mayo Clinic Study of Aging aged between 70 and 90 years showed that the effect of CVD, operationalized as white matter hyperintensities and brain infarcts, and Aβ on cognition was additive [10]. Similarly, a clinicopathological study of 148 community-dwelling older adults observed that after accounting for Aβ and tau, subcortical infarcts further lowered cognitive performance and increased the odds of progression to dementia by approximately 5 times [11]. More recently, a study of 63 older adults enrolled in the Alzheimer’s Disease Neuroimaging Initiative (ADNI) who consented to autopsy observed that higher degrees of arteriosclerosis was associated with greater neurofibrillary tau tangles, even after accounting for the extent of Aβ plaques [12]. When tested in an experimental mouse model, cerebral hypoperfusion increased white matter ischaemic damage which, in turn, induced increased tau tangle formation [12], providing experimental support for a relationship between CVD and tau, independent of Aβ [13, 14]. 

Together, converging evidence suggests that concurrent AD and CVD pathophysiological processes likely independently lower the threshold for cognitive decline and, subsequently, clinically classified dementia [7, 15]. However, most studies operationalize CVD using only a single pathological marker of tissue damage (e.g., micro- or macro-infarcts) or vessel disease (e.g., white matter hyperintensities) [16]. Further, few studies have examined the relationship between tau and concurrent CVD on cognitive performance and decline, and this requires additional clarification. Finally, many *in vivo* longitudinal studies such as ADNI and the Australian Imaging, Biomarkers and Lifestyle (AIBL) study have strict study inclusion criteria which excludes individuals with cardio- or cerebrovascular disease [17, 18]. Given that CVD is present in approximately 50–84% of individuals aged 80–90 + at autopsy [15, 19, 20], and is associated with an increased risk of progression to AD and vascular dementia, even after accounting for levels of Aβ and tau [7, 21–24], it is important to understand how hallmark AD biomarkers relate to cognition in initially CU individuals with concurrent CVD.

This study aimed to determine *ante-mortem* cognitive performance and trajectories associated with abnormalities in Aβ and/or tau at autopsy over 10 years in initially CU older adults with concurrent CVD. The first hypothesis was that autopsy-classified A + T + older adults would demonstrate poorer cognitive performance at the last visit before death and the fastest rate of cognitive decline in the years preceding death compared to older adults classified as A − T− or A + T−. The second hypothesis was that A − T + older adults would demonstrate poorer cognitive performance at the last visit before death and faster cognitive decline in the years preceding death compared to the A − T− group.

## Methods

### Data source

Data used in this study were obtained from the National Alzheimer’s Coordinating Center (NACC) database. The NACC initiative gathers data from a network of Alzheimer’s Disease Research Centers (ADRCs) located across the United States. Participants complete clinical evaluations annually according to a standardized protocol, with data collected forming the Uniform Data Set (UDS) [25]. Data collected include a detailed demographic assessment, physical/neurological exams, a neuropsychological assessment battery, questionnaires assessing neuropsychiatric symptoms, family health history, functional abilities, medical history, and medication use. Participants also underwent apolipoprotein E (*APOE* ε4) genotyping. The UDS has been described in detail previously [26, 27]. A subset of participants who consent to autopsy undergo a standardized neuropathological examination, forming the standardized Neuropathology Data Set (NPDS). This study included data from 28 ADRCs collected from September 2005 to March 2015. Importantly, NACC conducts quarterly data freezes; therefore, the data used in this study was specific to the September 2021 data freeze and the eligibility criteria upon which the datafile was created. To maximise data consistency, a small number of participants who had initial assessments using the C2 neuropsychological battery (implemented in NACC from March 2015) [28] were excluded at the datafile set-up stage.

### Ethics approval and consent to participate

The University of Washington Institutional Review Board approves all research using the NACC database and ethical approval was also obtained from individual ADRC’s Institutional Review Boards in accordance with the ethical standards of the Declaration of Helsinki. Written informed consent was obtained from all participants and informants prior to any study procedures.

### Participants

**Fig. 1:**
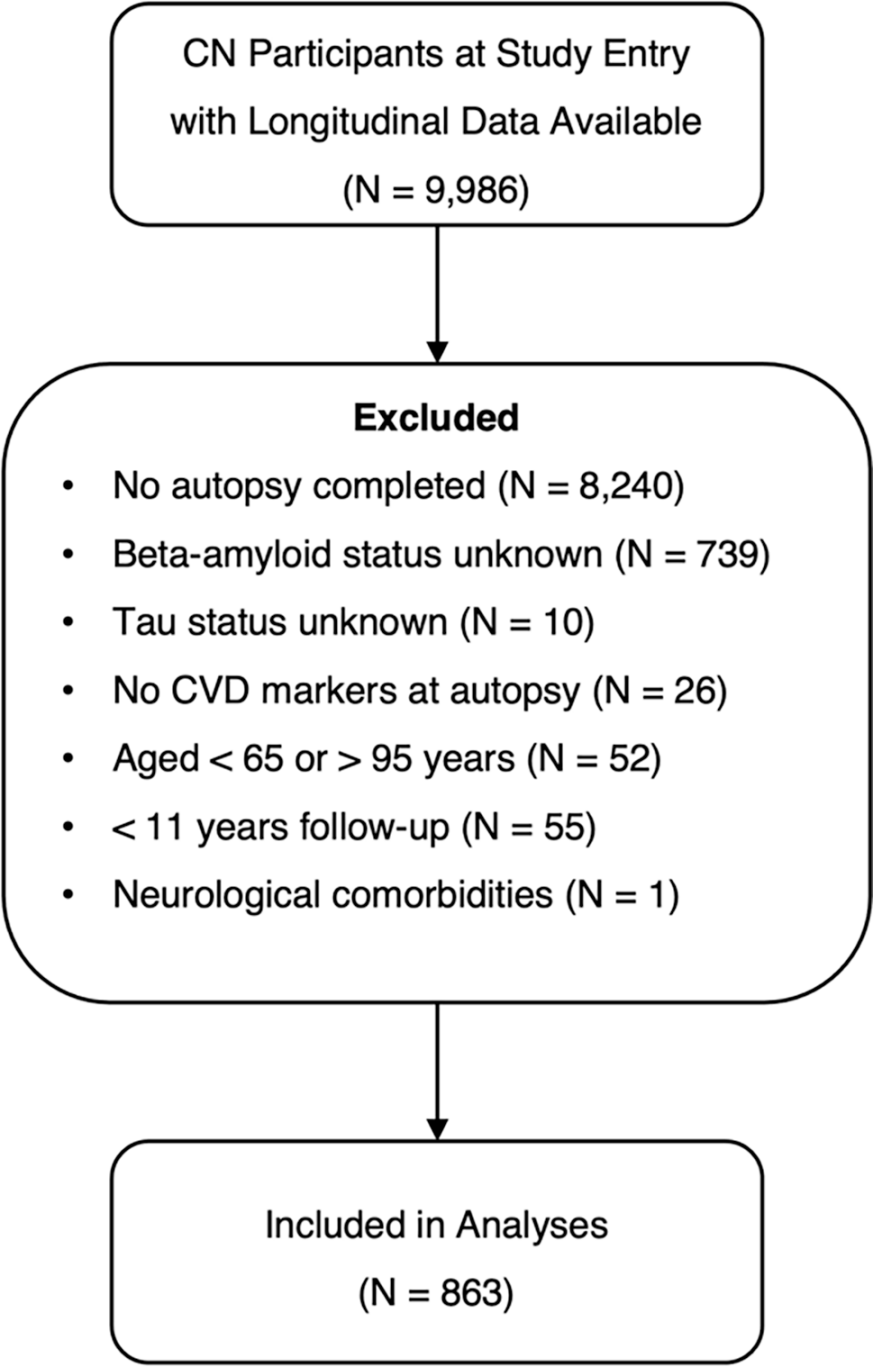
Flow-chart demonstrating the inclusion/exclusion criteria used to obtain the sample of participants included this study

Figure 1 details the inclusion/exclusion of participants in this study. Participants from NACC (*n* = 863) were included in this study if they were classified as CU at study entry, were aged between 65 and 95 years of age, had at least one longitudinal follow-up, had sufficient autopsy data available, *APOE* ε4 genotype was known, were present in the NPDS, and had autopsy evidence of at least one out of six markers of CVD. Participants were classified as CU at study entry if an expert clinical panel classified them as having “normal” cognition [29]. Participants with a dominantly inherited AD mutation (*N* = 1) or severe neurological or neuropsychiatric conditions (excluding MCI or dementia) with potential to influence cognitive outcomes (see Additional File 1 for full list) were excluded from this analysis. The sample was also restricted to include those < 11 years from the time of death, due to very limited data at follow-up timepoints thereafter.

### Neuropsychological assessments

A modified version of the Preclinical Alzheimer’s Cognitive Composite (PACC) was constructed using the Mini-Mental Status Examination (MMSE), Logical Memory Delayed Recall (dMemory), and the Digit Symbol Substitution Test (DSST) from the Wechsler Adult Intelligence Scale – Revised. The PACC computation typically includes two measures of delayed memory recall; however, because only one delayed memory measure was available in NACC, the dMemory was given twice the weight to reflect the contribution of delayed memory recall in the composite, following a previously published method [30]. PACC score was computed by first standardizing raw scores for each outcome measure using the baseline mean and standard deviation of the current sample, and then averaging the standardized MMSE, dMemory (2x weighting), and DSST scores. A composite measure of episodic memory was generated by computing standardized z-scores for both the Immediate and Delayed Recall Subtests of the Wechsler Memory Scale-Revised (scores on both subtests range from 0 to 25) using the baseline mean and standard deviation of the current sample, and then averaging the standardized scores. A composite measure of executive function was generated by computing standardized z-scores for Digit Span Backwards (total correct), Trail Making Test parts A and B (correct lines per minute), lexical fluency (F and L words – total correct), and semantic fluency (animal and vegetable fluency – total correct) using the baseline mean and standard deviation of the current sample, and then averaging the standardized scores [31]. 

### *APOE* ε4 genotype

*APOE* ε4 carriage vs. non-carriage was classified according to the number of ε4 alleles (1 or 2 ε4 alleles = ε4+, 0 ε4 alleles = ε4-).

### Autopsy neuropathological methods and measures

Neuropathological examinations were performed using standardized methods at each centre in accordance with relevant guidelines and regulations, with data collected via a standardized neuropathology form and coding guidebook, described in detail previously [32]. 

### Aβ plaque and Tau tangle staging at autopsy

Participants were classified into A − T−, A + T−, A − T + or A + T + based on semiquantitative Consortium to Establish a Registry for Alzheimer’s Disease (CERAD) neuritic plaque staging and Braak staging. CERAD neuritic plaque staging was used to classify participants as A+ (moderate or frequent plaque deposition) or A− (no or sparse plaque deposition) at autopsy [33]. Semiquantitative Braak neurofibrillary tangle staging was used to classify participants as abnormal for tau pathology (T+; Braak stages III-VI) or tau negative (T−; Braak stages 0-II) at autopsy [33]. 

### CVD classification at autopsy

CVD at autopsy was classified according to the presence of absence of large artery infarcts or lacunes (old/acute/subacute), haemorrhages (old/acute/subacute), microinfarcts (old/acute/subacute), subcortical arteriosclerotic leukoencephalopathy, atherosclerosis of the Circle of Willis and arteriolosclerosis, following a recently described method [34]. If the CVD marker was listed as ‘present’ at autopsy, participants were assigned a score of ‘1’ and scores were summed to form a total score ranging from 0 to 6, with higher scores indicative of greater CVD burden. Only participants with a score of ≥ 1 were included in the analysis.

### Data analysis

All analyses and visualisations were conducted in RStudio using the R program for statistical computing (version 3.6.1), using the following packages: “lme4”, “lmerTest”, “dplyr”, “ggplot2”, “emmeans”, and “effects”.

Differences in demographic, clinical, neuropsychological, and Aβ, tau, and CVD pathology variables at study entry and/or the last visit before death were assessed across autopsy pathology groups using a series of analyses of variance for continuous variables and chi-square tests for categorical variables, including pairwise comparisons where applicable.

Neuropsychological data from the last visit before death were used for all cross-sectional analyses. A series of analyses of covariance (ANCOVA) with pairwise comparisons first explored associations between pathology status at autopsy (A − T−, A + T−, A − T+, A + T+) and performance on the PACC, episodic memory, and executive function composites at the last visit before death, controlling for age, sex, *APOE* ε4, the interval between death and the last study visit, and cognitive status at the last visit before death (CU, MCI, dementia). Estimated marginal means (EMMs) were calculated for each group, to compute measures of effect size (Cohen’s d) between groups, with values of 0.2, 0.5 and 0.8 indicative of small, medium, and large effects, respectively [35]. Clinical diagnoses across autopsy pathology groups at the last visit before death were determined using chi-square tests and both odds ratios and 95% confidence intervals were computed.

Longitudinal models leveraged all available neuropsychological data to evaluate cognitive trajectories before death, with time modelled as the visit number for each row minus the total number of all visits. Time ‘0’ was indicative of the last visit before death. Reverse-time linear mixed effects models (LMM) assessed two-way interactions between autopsy pathology status (A − T−, A + T−, A − T+, A + T+) × time on the rate of change in PACC, episodic memory, and executive function. A series of planned comparisons were conducted to determine differences in *ante-mortem* cognitive trajectories between groups of interest: [1] A − T− vs. A + T+, to characterise the nature and magnitude of Aβ and tau pathology on cognitive decline [2], A + T − vs. A + T+, to characterise the nature and magnitude of tau pathology on cognitive decline in the presence of Aβ pathology, and [3] A − T− vs. A − T+, to characterise the nature and magnitude of tau pathology on cognitive decline in the absence of Aβ pathology. Interactions between covariates (age, sex, *APOE* ε4, self-or informant reported stroke, self- or informant reported transient ischemic attack) × time were included as separate predictors and the interval between death and the last study visit was also added as a covariate. EMMs and standard error were computed for each group, and the magnitude of difference in rates of change between groups was expressed using Cohen’s d effect sizes. The level of probability required for classification of statistical significance was set at *p* <.05 as only three outcomes were considered and estimates of magnitude (Cohen’s d) were used to contextualize results.

### Data Availability

NACC data is available for use upon written request (https://naccdata.org/requesting-data/data-request-process). Authors who access the data are required to sign and comply with a data use agreement.

## Results

### Demographic, cardiovascular risk, and neuropsychological characteristics at study entry and the last visit before death (cross-sectional baseline)

Tables 1 and 2 summarize the demographic, cardiovascular risk, and neuropsychological characteristics of the sample at study entry and the last visit before death stratified across autopsy groups. On average, participants underwent 5.1 ± 2.6 study visits and started their first assessment 7.2 ± 3.2 years prior to death. At study entry, participants had a mean age of 81.8 years (± 6.9), 62% were female, and 27.5% were *APOE* ε4 carriers.

At the last visit before death, mean age was 87.2 years (± 6.5) and age differed significantly across groups, with the A − T + group being the oldest (Table 2). Performance on the MMSE differed significantly across groups at the last visit before death, with A + T + adults demonstrating the poorest performance, followed by the A − T+, A + T−, and A − T− groups. Groups also differed significantly across several indicators of cardiovascular health, including body mass index (BMI), systolic blood pressure, and hypercholesterolemia (Table 2).

**Table 1:** Demographic, risk and neuropsychological characteristics of the sample at study entry

Study entry	Total Sample
Age (M, SD, range)	81.8 (6.9), 65–95
Sex (n, % Female)	328/535 (62%)
Education (Years) (M, SD)	15.7 (2.9)
Ethnicity (n, %)	
*White*	819 (95.1%)
*Black/African American*	37 (4.3%)
*Asian*	3 (0.4%)
*American Indian/Alaska Native*	1 (0.1%)
*Native Hawaiian/Other Pacific Islander*	1 (0.1%)
*Other*	0(0%)
GDS Score (M, SD)	1.7 (3.7)
BMI (M, SD)	26.2 (4.7)
Systolic Blood Pressure (M, SD)	135.4 (18.5)
Hypertension (n, %)	343/520 (60.3%)
Diabetes (n, %)	772/90 (10.4%)
Hypercholesterolemia (n, %)	465/391 (46%)
Stroke hx (n, %)	
*Absent*	822 (95.5%)
*Recent/Active*	6 (0.7%)
*Remote/Inactive*	33 (3.8%)
TIA hx (n, %)	
*Absent*	785 (91.4%)
*Recent/Active*	27 (3.1%)
*Remote/Inactive*	47 (5.5%)
MMSE (M, SD)	28.7 (1.4)
Episodic Memory (M, SD)	-0.00 (1.0)
PACC (M, SD)	0.00 (0.7)
Executive Function (M, SD)	0.00 (0.7)
*APOE ε4* Carrier (n, %)	626/237 (27.5%)

Note. Complete cardiovascular risk factor data not available for all participants for some variables (e.g., hypercholesterolemia, total *n* = 856), thus, percentages calculated from total n available. n/n (%) values for sex first present the number of males, then the number of females, followed by the proportion of participants (%) classified as female. n/n (%) values for hypertension, diabetes, hypercholesterolemia, and *APOE* ε4 status first present the number of participants without the risk factor, then the number of participants with the risk factor, followed by the proportion of participants (%) with the risk factor. Abbreviations: M = mean, SD = standard deviation, GDS = Geriatric Depression Scale, BMI = body mass index, TIA = transient ischemic attack, MMSE = Mini-Mental State Examination, PACC = Preclinical Alzheimer’s Cognitive Composite, *APOE* = Apolipoprotein

**Table 2:** Demographic, risk and neuropsychological characteristics of the sample at the last visit before death and autopsy measures of Aβ, tau, and cerebrovascular disease stratified by autopsy status (A − T−, A + T−, A − T+, A + T+)

Last visit before death	N Available	Total Sample	A − T+	A + T−	A − T+	A + T+	*p*
n autopsy status (% of sample)		-	176 (20%)	128 (15%)	157 (18%)	402 (47%)	-
**Age (M, SD, range)**	863	87.2 (6.5), 67–95	85.1 (7.2), 67–95	84.4 (6.8), 68–95	89.8 (5.3), 70–95	88.0 (5.9), 69–95	**< .000**
**Sex (n, % Female)**	863	328/535 (62%)	85/91 (51.7%)	50/78 (60.9%)	53/104 (66.2%)	140/262 (65.2%)	**0.01**
**Education (Years) (M, SD)**	856	15.7 (2.9)	15.6 (3.1)	15.8 (2.8)	15.4 (2.7)	15.8 (2.9)	0.43
**GDS Score (M, SD)**	863	3.4 (12.8)	3.1 (11.6)	3.0 (11.2)	3.6 (12.3)	3.7 (14.0)	0.92
**BMI (M, SD)**	639	25.1 (4.9)	25.6 (5.4)	26.5 (5.9)	25 (4.4)	24.5 (4.4)	**0.003**
**Systolic Blood Pressure (M, SD)**	681	131.9 (19.1)	128.7 (19.3)	131.1 (17.4)	131.0 (19.2)	134.2 (19.4)	**0.03**
**Hypertension (n, %)**	600	161/439 (73.2%)	30/91 (74.4%)	33/91 (73.4%)	20/74 (78.7%)	78/183 (70.1%)	0.4
**Diabetes (n, %)**	598	530/68 (11.4%)	108/12 (10%)	104/19 (15.4%)	79/15 (16%)	239/22 (8.4%)	0.09
**Hypercholesterolemia (n, %)**	589	268/321 (54.5%)	54/65 (55%)	34/60 (64%)	72/47 (39.5%)	108/149 (58%)	**0.002**
**Stroke (n, %)**	598						
*Absent*	-	527 (88.1%)	106 (88.3%)	84 (89.4%)	103 (83.7%)	**234 (89.7%)**	**< .000**
*Recent/Active*	-	16 (2.7%)	**5 (4.2%)**	2 (2.1%)	4 (3.3%)	5 (1.9%)	0.68
*Remote/Inactive*	-	55 (9.2%)	9 (7.5%)	8 (8.5%)	**16 (13%)**	22 (8.4%)	**0.02**
**TIA (n, %)**	593						
*Absent*	-	496 (83.6%)	101 (84.2%)	76 (81.7%)	**106 (86.9)**	213 (82.6%)	**< .000**
*Recent/Active*	-	22 (3.7%)	4 (3.3%)	**6 (6.5%)**	5 (4.1%)	7 (2.7%)	0.82
*Remote/Inactive*	-	75 (12.6%)	15 (12.5%)	11 (11.8%)	11 (9%)	**38 (14.7%)**	**< .000**
**MMSE (M, SD, range)**	588	26.8 (4.3), 1–30	28.2 (2.7), 8–30	28.0 (2.4), 18–30	27.2 (2.9), 16–30	25.6 (5.4), 1–30	**< .000**
**Diagnosis (n, %)**	863						
*Cognitively normal*	-	480 (55.6%)	**136 (77.2%)**	97 (75.8%)	97 (61.8%)	150 (37.3%)	**0.002**
*Impaired/MCI*	-	176 (20.4%)	19 (10.8%)	13 (10.2%)	36 (22.9%)	**108 (26.9%)**	**< .001**
*Dementia*	-	207 (24%)	21 (12%)	18 (14%)	24 (15.3%)	**144 (35.8%)**	**< .001**
**Years from last visit to death (M, SD)**	863	1.82 (1.9)	1.45 (1.5)	1.53 (1.6)	2.1 (2.1)	2.0 (2.0)	**0.002**
**Determined at Autopsy**		**Total Sample**	**A** − **T**−	**A + T**−	**A** − **T+**	**A + T+**	***p***
**CERAD Score (M, SD)**	863	1.8 (1.2)	0.4 (0.5)	2.5 (0.5)	0.5 (0.5)	2.7 (0.5)	**< .000**
**Braak Stage (M, SD)**	863	3.3 (1.5)	1.5 (0.6)	1.6 (0.5)	3.8 (0.8)	4.4 (1.0)	**< .000**
**CVD Burden Score (M, SD)**	863	2.9 (1.3)	3.0 (1.4)	2.9 (1.3)	3.1 (1.3)	2.8 (1.3)	0.07

Note. Complete demographic/cardiovascular risk factor data not available for all participants for some variables, thus, percentages for the total sample were calculated using the total n available (shown in ‘N Available’ column). Percentages for the autopsy status groups were calculated from the total n available in each group (e.g., for hypertension, 91/121 (total n available data) * 100). n/n (%) values for sex first present the number of males, then the number of females, followed by the proportion of participants (%) classified as female. n/n (%) values for hypertension, diabetes, and hypercholesterolemia first present the number of participants without the risk factor, then the number of participants with the risk factor, followed by the proportion of participants (%) with the risk factor. Abbreviations: M = mean, SD = standard deviation, GDS = Geriatric Depression Scale, BMI = body mass index, TIA = transient ischemic attack, MMSE = Mini-Mental State Examination, MCI = mild cognitive impairment, CERAD = Consortium to Establish a Registry for Alzheimer’s Disease, CVD = cerebrovascular disease

### Measures of Aβ, tau, and CVD at autopsy

The proportion of participants (in the order of highest prevalence) classified into each autopsy group was as follows: 47% A + T+, 20% A − T−, 18% A − T+, and 15% A + T− (Table 2). As expected, groups demonstrated significant differences in CERAD semi-quantitative scores and BRAAK staging scores (all *p*’s < 0.05), with the A + T + group demonstrating the highest CERAD semi-quantitative and Braak staging scores (see Table 2 and Additional File 1 (Table 1) for all pairwise comparisons, including Tukey-adjusted *p* values). At autopsy, the A − T + group had the highest mean CVD burden score, however, CVD burden did not differ significantly between groups (*p* =.07) (Table 2).

### Cross-sectional associations between autopsy status and PACC, episodic memory, and executive function at the last visit before death

**Fig. 2:**
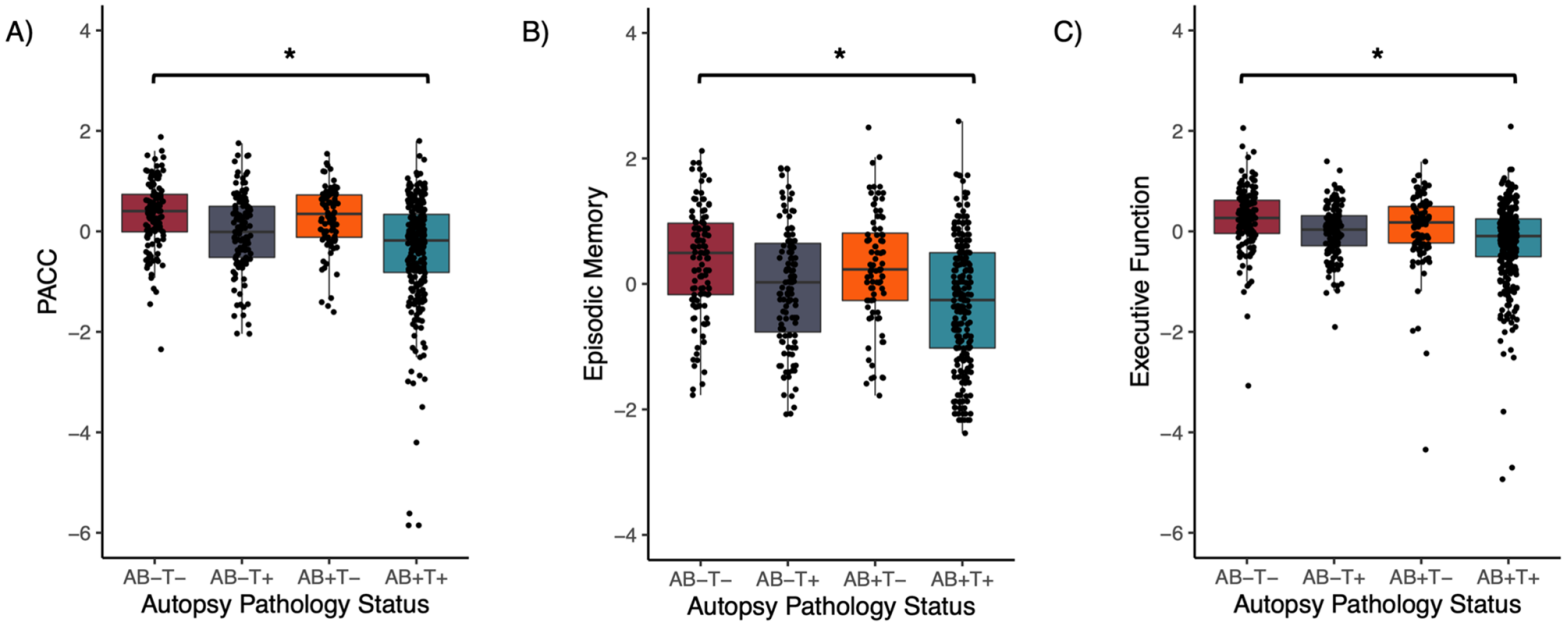
Distribution of cognitive scores by autopsy groupings. Note. Dot-boxplots showing cognitive performance at the last visit before death on the (**A**) PACC, (**B**) episodic memory, and (**C**) executive function composites by autopsy group. A − T− is denoted in maroon, A − T + is denoted in navy, A + T − is denoted in orange and A + T + is denoted in turquoise. Values on the y-axis that progressively become more negative reflect poorer cognitive performance. * denotes statistical significance at *p* <.05. Abbreviations: PACC = Preclinical Alzheimer’s Cognitive Composite

Table 3 summarizes the associations between autopsy status and performance on the PACC, episodic memory, and executive function composites. A series of ANCOVAs indicated that groups differed significantly on all cognitive composites (all *p*’s < 0.05). Specifically, at the last visit before death, when compared to the A − T− group, the A + T + group performed significantly worse on the PACC, episodic memory, and executive function composites, although the magnitude of difference between groups was small (Cohen’s d’s = 0.23–0.24) (see Fig. 2 and Additional File 1 (Tables 2, 3 and 4) for pairwise comparisons). At the last visit before death, performance on all cognitive composites was equivalent between the A − T−, A − T + and A + T − groups.

**Table 3:** Performance on the episodic memory, PACC, and executive function composites at the last visit before death (cross-sectional baseline) stratified by autopsy status (A − T−, A + T−, A − T+, A + T+)

				Autopsy Status			
**Last visit before death**	**Group differences**			**A** − **T**− **(** *n* ** = 176)**	**A + T**− **(** *n* ** = 128)**	**A** − **T+** **(** *n* ** = 157)**	**A + T+** **(** *n* ** = 402)**
	***F*** **-ratio (df)**	***η***p^**2**^	***p***	**EMM (SE)**	**EMM (SE)**	**EMM (SE)**	**EMM (SE)**
Episodic Memory	23.7 (3)	0.11	**< 0.000**	0.11 (0.08)	-0.06 (0.09)	-0.11 (0.08)	-0.14 (0.05)
PACC	35.6 (3)	0.15	**< 0.000**	0.07 (0.07)	-0.07 (0.08)	-0.09 (0.07)	-0.19 (0.08)
Executive Function	20.7 (3)	0.08	**< 0.000**	0.07 (0.06)	-0.11 (0.06)	-0.04 (0.06)	-0.10 (0.04)

Abbreviations: ηp2 = partial eta squared, EMM = estimated marginal mean, SE = standard error, PACC = Preclinical Alzheimer’s Cognitive Composite

### Clinical status across autopsy groups at the last visit before death

At the last visit before death, a significantly greater proportion of adults classified as A + T + at autopsy (35.8%) received a clinical diagnosis of dementia compared to the A − T− (12%; OR = 6.86), A + T− (14%; OR = 8.00), and A − T+ (15.3%; OR = 6.00) groups (Tables 4 and 5). Similarly, a significantly greater proportion of adults classified as A + T + at autopsy received a clinical diagnosis of cognitive impairment/MCI compared to the A − T− (10.8%; OR = 5.68), A + T− (10.2%; OR = 8.31), and A − T+ (22.9%; OR = 3.00) groups (Tables 4 and 5). Clinical diagnostic rates at the final visit before death for both MCI and dementia were 44.4% in total.

**Table 4:** Clinical diagnoses at the last visit before death stratified by autopsy status (A − T−, A + T−, A − T+, A + T+)

Last visit before death	Full Sample	A − T−	A + T−	A − T+	A + T+	*p*
	(n = 863)	(n = 176)	(n = 128)	(n = 157)	(n = 402)	
Diagnosis (n, %)						
***Cognitively normal***	480 (55.6%)	**136 (77.2%)**	97 (75.8%)	97 (61.8%)	150 (37.3%)	**< .001**
***Impaired/MCI***	176 (20.4%)	19 (10.8%)	13 (10.2%)	36 (22.9%)	**108 (26.9%)**	**< .001**
***Dementia***	207 (24%)	21 (12%)	18 (14%)	24 (15.3%)	**144 (35.8%)**	**< .001**

Note. Impaired-not-MCI and MCI groups were combined due to the small sample size of participants classified as cognitively impaired not MCI (*N* = 13). Percentages shown reflect within-group proportion of participants who received a diagnosis (e.g., proportion of A − T− adults classified as CN, MCI or dementia). *p* reflects between-group differences in the proportion of participants who received a diagnosis by autopsy status. Bolding highlights the group with the highest proportion of participants (between-group) who received a diagnosis. Abbreviations: MCI = mild cognitive impairment

**Table 5:** Proportion of participants who received a clinical diagnosis across comparison groups with odds ratios

Last visit before death	A + T+	A − T−	OR [95% CI]
**Group**			
*Cognitively normal* *Impaired/MCI* *Dementia*	150 (37.3%) 108 (26.9%) 144 (35.8%)	136 (77.2%) 19 (10.8%) 21 (12%)	1.10 [0.87–1.39] 5.68 [3.49–9.23] 6.86 [4.34–10.84]
	**A + T+**	**A + T-**	**OR [95% CI]**
*Cognitively normal* *Impaired/MCI* *Dementia*	150 (37.3%) 108 (26.9%) 144 (35.8%)	97 (75.8%) 13 (10.2%) 18 (14%)	1.55 [1.20–2.00] 8.31 [4.67–14.77] 8.00 [4.90–13.06]
	**A − T−**	**A − T+**	**OR [95% CI]**
*Cognitively normal* *Impaired/MCI* *Dementia*	136 (77.2%) 19 (10.8%) 21 (12%)	97 (61.8%) 36 (22.9%) 24 (15.3%)	1.40 [1.08–1.82] 0.53 [0.30–0.92] 0.88 [0.49–1.57]

Note. Impaired-not-MCI and MCI groups were combined due to the small sample size of participants classified as cognitively impaired not MCI (*N* = 13). Interpretation of OR’s is as follows: OR = 1: odds of the outcome are the same in both groups; OR > 1: odds of the outcome (clinical diagnosis) are higher in the group listed in the first column compared to the group listed in the second column; OR < 1: odds of the outcome are lower in the group listed in the first column compared to the group listed in the second column. Abbreviations: OR = odds ratio, CI = confidence interval, MCI = mild cognitive impairment

### Longitudinal associations between autopsy status and *ante-mortem* cognitive trajectories

**Fig. 3:**
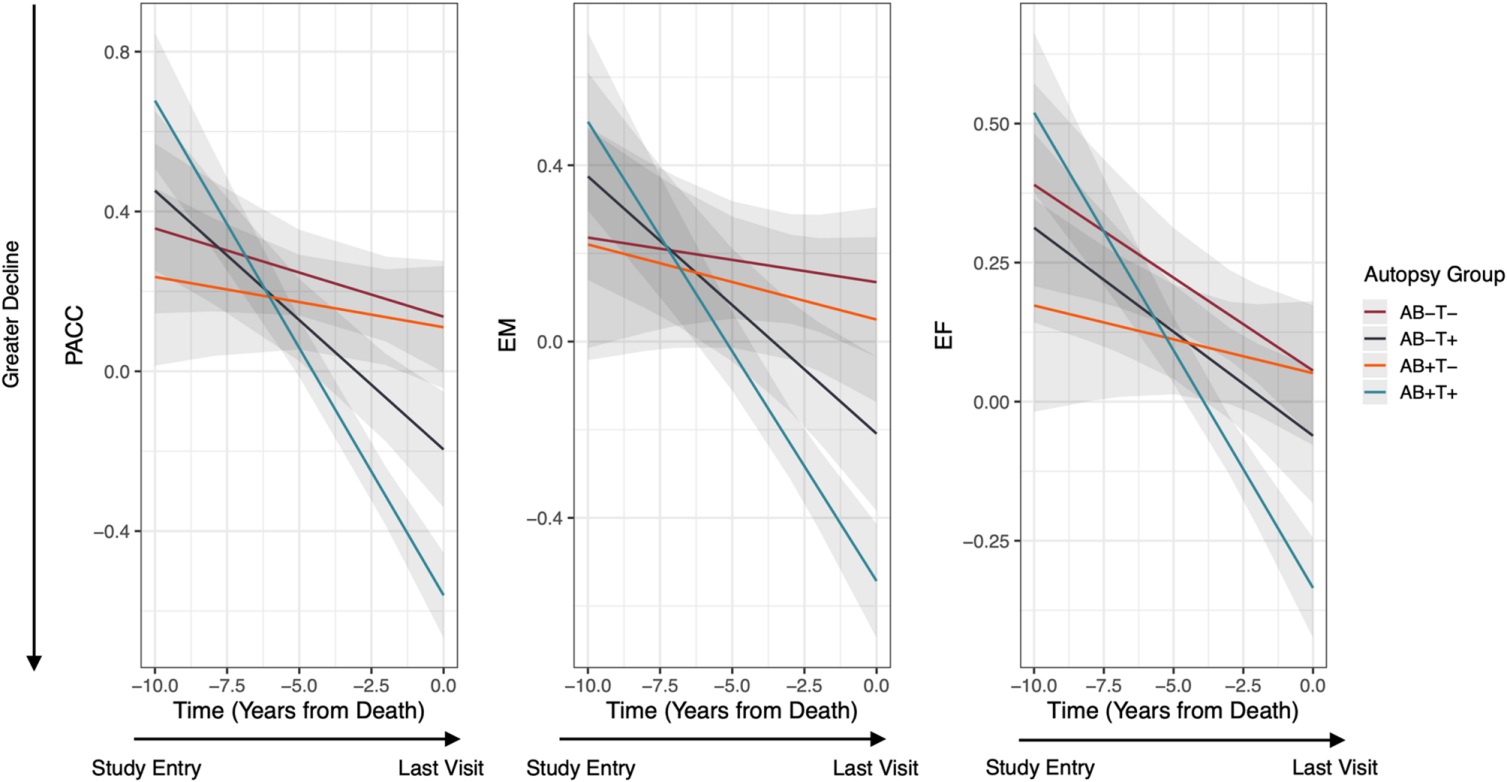
Rates of *ante-mortem* cognitive change across autopsy groups. Note. Figure depicts rates of *ante-mortem* cognitive change across groups (A − T− (red line); A − T+ (black line); A + T− (orange line); A + T+ (blue line)) over time, after accounting for age × time, sex × time, *APOE* ε4 × time, self- or informant reported stroke × time, and self- or informant reported TIA × time interactions, and the number of years between the last study visit and death. Reverse-time linear mixed models compared effects between the A − T− vs. A + T + groups, the A + T − vs. A + T + groups, and the A − T− vs. A − T + groups. Higher (positive) scores on the y-axis (PACC, EM, EF) are indicative of better PACC, EM, and EF performance. ‘Time’ was modelled as the visit number for each row minus the total number of all visits made, with time ‘0’ indicative of the last visit before death. Shading indicates 95% CIs. Abbreviations: PACC = Preclinical Alzheimer’s Cognitive Composite, EM = episodic memory, EF = executive function

In the ~ 10 years preceding death, and when compared to A − T− adults, A + T + adults showed significantly faster decline on the PACC (d = 0.46), episodic memory (d = 0.37), and executive function (d = 0.34) composites, with the magnitude of decline, by convention, small-to-moderate (Table 6; Fig. 3). Similarly, when compared to A + T − adults, A + T + adults showed significantly faster decline on the PACC (d = 0.37), episodic memory (d = 0.31), and executive function (d = 0.19) composites, with the magnitude of decline, by convention, very small-to-moderate (Table 6; Fig. 3). Lastly, when compared to A − T− adults, A − T + adults showed significantly faster decline on the PACC (d = 0.25) and episodic memory (d = 0.18) composites, although the magnitude of difference between groups on episodic memory was very small (< 0.2) (Table 6; Fig. 3). A − T− and A − T + groups did not differ on rates of change on the executive function composite, and the magnitude of difference between groups was also very small (d = 0.18, *p* =.72).

### Longitudinal interactions between autopsy status and time on *ante-mortem* cognitive trajectories in older adults without dementia at the last visit before death

To assess the extent to which the longitudinal effects were being driven by *ante-mortem* clinical diagnostic status, a sensitivity analysis was conducted excluding participants with a diagnosis of dementia at the last visit before death. When analyses were restricted to include older adults without dementia, significantly faster *ante-mortem* cognitive decline on all outcomes was similarly observed in A + T + adults compared to A − T− adults in the ~ 10 years preceding death (d = 0.37, 0.32 and 0.29 for PACC, episodic memory, and executive function, respectively; Table 6). Similarly, when compared to A + T − adults, A + T + adults showed significantly faster decline on all cognitive composites (d = 0.28, 0.26 and 0.10 for PACC, episodic memory, and executive function, respectively), although the magnitude of difference between groups was reduced (Table 6). Lastly, when compared to A − T− adults, A − T + adults also showed significantly faster decline on PACC (d = 0.27) and episodic memory (d = 0.19) composites, but not the executive function composite (d = 0.21, *p* =.76) (Table 6).

**Table 6:** Summary of results from the reverse-time linear mixed models (longitudinal) exploring two-way interactions between autopsy groups (A − T−, A + T−, A − T+, A + T+) × time on rates of *ante-mortem* cognitive change across the total sample and those without dementia

All Diagnoses	Outcomes								
	**PACC**			**Episodic Memory**			**Executive Function**		
**Model Predictors (Term)**	**β Estimate**	**SE**	***p***	**β Estimate**	**SE**	***p***	**β Estimate**	**SE**	***p***
Time	1.38	1.53	0.47	7.16	1.68	**< 0.001**	-3.82	1.37	**0.005**
*APOE* ε4	-1.98	4.94	**< 0.001**	-2.54	6.24	**< 0.001**	-9.16	4.04	**0.02**
Age	-1.73	3.15	**< 0.001**	-8.68	3.99	**0.03**	-1.60	2.60	**< 0.001**
Sex	1.10	4.35	**0.01**	1.79	5.50	**0.001**	7.93	3.57	**0.03**
Stroke	1.24	3.18	**< 0.001**	-3.89	3.61	0.28	-1.22	2.75	**< 0.001**
TIA	-4.24	2.59	0.87	-8.51	2.97	0.98	2.19	2.23	0.33
Age × Time	-3.49	1.70	**0.04**	-8.84	1.88	**< 0.001**	3.39	1.53	**0.03**
Sex × Time	-3.84	2.03	0.06	-7.72	2.22	**< 0.001**	-8.73	1.82	**< 0.001**
*APOE* ε4 × Time	-1.02	2.29	**< 0.001**	-1.02	2.50	**< 0.001**	-5.59	2.06	**0.007**
Stroke × Time	6.69	2.26	**0.003**	7.07	2.44	**0.004**	-2.68	2.03	0.19
TIA × Time	9.13	1.76	0.60	1.92	1.91	0.32	4.51	1.57	0.77
A − T− vs. A + T+ × Time	-2.69	2.85	**< 0.001**	-2.49	3.12	**< 0.001**	-1.38	2.56	**< 0.001**
A + T − vs. A + T+ × Time	-2.95	3.05	**< 0.001**	-2.31	3.34	**< 0.001**	-1.94	2.75	**< 0.001**
A − T− vs. A − T+ × Time	-1.13	3.34	**0.001**	-1.28	3.65	**0.000**	-1.05	2.99	0.72
**Without Dementia**	**Outcomes**								
	**PACC**			**Episodic Memory**			**Executive Function**		
**Model Predictors (Term)**	**β Estimate**	**SE**	***p***	**β Estimate**	**SE**	***p***	**β Estimate**	**SE**	***p***
Time	5.67	1.18	**< 0.001**	9.36	1.67	**< 0.001**	-2.11	1.11	0.06
*APOE* ε4	-1.47	4.28	**0.001**	-2.04	6.00	**0.001**	-5.10	3.65	0.16
Age	-1.58	2.70	**< 0.001**	-6.67	3.82	0.08	-1.52	2.32	**< 0.001**
Sex	1.35	3.77	**< 0.001**	2.10	5.28	**< 0.001**	1.08	3.21	**< 0.001**
Stroke	-5.83	2.61	**0.03**	-2.12	3.64	0.95	-8.88	2.38	**< 0.001**
TIA	1.28	2.07	0.95	-2.00	2.91	0.49	8.73	1.89	0.64
Age × Time	-7.22	1.31	**< 0.001**	-1.08	1.85	**< 0.001**	2.21	1.24	0.07
Sex × Time	-8.91	1.55	0.57	-3.84	2.19	0.08	-6.22	1.47	**< 0.001**
*APOE* ε4 × Time	-3.49	1.79	**0.05**	-2.26	2.53	0.37	2.03	1.69	0.23
A − T− vs. A + T+ × Time	-1.61	2.13	**< 0.001**	-1.91	3.02	**< 0.001**	-8.57	2.02	**< 0.001**
A + T − vs. A + T+ × Time	-1.67	2.31	**< 0.001**	-1.57	3.27	**< 0.001**	-1.14	2.19	**< 0.001**
A − T− vs. A − T+ × Time	-9.08	2.47	**< 0.001**	-1.15	3.50	**0.001**	-7.15	2.35	0.76

Note. All participants classified as cognitively unimpaired at study entry. Reverse-time linear mixed models compared effects between the A − T− vs. A + T + groups (A − T− as the reference group), the A + T − vs. A + T + groups (A + T − as the reference group), and the A − T− vs. A − T + groups (A − T− as the reference group). All other model estimates were obtained from the model using the A − T− group as the reference group. Abbreviations: PACC = Preclinical Alzheimer’s Cognitive Composite, β = beta, SE = standard error, *APOE* ε4 = Apolipoprotein E ε4

## Discussion

This study aimed to determine *ante-mortem* cognitive performance and trajectories associated with abnormal levels of Aβ/tau at autopsy in a sample of initially cognitively unimpaired older adults with concurrent CVD. The first hypothesis that older adults classified as A + T + at autopsy would demonstrate the poorest cognitive performance at the last visit before death, and the fastest cognitive decline in the years preceding death compared to A − T− or A + T − adults was supported. At the last visit before death, A + T + adults with autopsy-confirmed CVD demonstrated the poorest cognitive performance (Table 3). Further, A + T + adults showed the fastest trajectory of cognitive decline, particularly when measured on the PACC, over a period of ~ 10 years *ante-mortem*, and this was approximately ten times greater than the decline observed in A − T− adults (Table 6; Fig. 3). These findings remained even when the sample was restricted to include only those without dementia at the last visit before death. However, despite the presence of significant Aβ and tau pathology, and the increased rate of cognitive decline *ante-mortem* in the A + T + group, final visit dementia diagnosis was low (35.8%), suggesting that even with high levels of mixed pathologies, progression to clinically classified dementia is not inevitable prior to death. The results of this study also indicate that the magnitude of difference in *ante-mortem* cognitive decline between the A − T− and A + T + groups was similar to the magnitude of difference observed between the A + T − and A + T + groups. This is consistent with, and extends, recent results showing a dominant association between amyloidosis and longitudinal memory decline, but only in the presence of tauopathy, neurodegeneration, or both [5]. 

The second hypothesis that A − T + older adults would demonstrate poorer cognitive performance and faster cognitive decline in the years preceding death compared to A − T− older adults was partially supported. A small difference in *ante-mortem* cognitive trajectories, but not performance, was observed on the PACC (d = 0.25). No differences in *ante-mortem* cognitive performance or trajectories between these groups were initially observed on the episodic memory and executive function composites (effect sizes < 0.2). However, when the sample was restricted to adults without dementia, A − T + adults demonstrated a faster rate of cognitive decline on the PACC and episodic memory composite compared to the A − T− group, although the difference in the rate of cognitive decline was only small in magnitude. Previous studies have generally shown that abnormal tau (e.g., elevated tau-PET, tau tangles at autopsy) is more strongly associated with increased cognitive impairment and decline than Aβ+ [36–39], even amongst cognitively unimpaired adults [5, 40]. However, given the small difference in cognitive decline between the A − T− and A − T + groups (e.g., d = 0.19 on episodic memory), compared to the larger difference in cognitive decline between the A + T − and A + T + groups without dementia (e.g., d = 0.26 on episodic memory), these results suggest that cognitive decline is amplified when abnormal tau occurs in the presence of extant amyloid plaque deposition [41–43]. Together, these findings are consistent with, and extend, recent in vivo studies showing that A + T + older adults without dementia demonstrate a steeper trajectory of cognitive decline over a period of 3.5 years compared to individuals with neither, or only one, abnormal AD pathology [44]. These findings also suggest that while concurrent pathophysiological processes (e.g., T+, CVD) can result in small impairments and declines in cognition, it is abnormality in Aβ, in combination with tau, that drives substantial cognitive decline.

Previous studies have examined associations between abnormal Aβ, tau, and neurodegeneration biomarkers, measured in vivo or via neuropathological series, on cognition in older adults with and without cognitive impairment. However, few have examined the manifestation of mixed pathologies on cognition, despite observations that pure AD is rare and most older adults present with at least some pathological marker of CVD at autopsy [45]. The finding that A + T + adults without dementia demonstrated the poorest cognitive performance and fastest rate of cognitive decline compared to A − T− and A + T − adults accords with and extends observations from recent cross-sectional and longitudinal studies. For example, our results showed that the A + T + group demonstrated the greatest cognitive impairment at the last visit before death and the fastest rate of *ante-mortem* cognitive decline, followed by the A + T − and A − T− groups. To reduce the potential for Type I error, formal statistical comparisons were only made between A − T− and A + T + adults, A + T − and A + T + adults, and A − T− and A − T + adults. However, examination of the pattern and trajectory of slopes illustrated in Fig. 3 suggests that A − T + adults likely demonstrate steeper cognitive declines compared to A + T − adults, but not A + T + adults, across all outcomes. This accords with a recent cross-sectional study in non-demented older adults enrolled in the Alzheimer’s Disease Neuroimaging Initiative (ADNI) which showed that while A + T + adults demonstrated the lowest cognitive performance across measures of memory, language and executive function [13], A − T + adults demonstrated small-to-moderate impairments in cognitive performance weaker in magnitude compared to A + T + adults, but stronger in magnitude compared to A − T− and A + T − adults [13]. 

The longitudinal results of this study are also consistent with a recent prospective in vivo study in initially cognitively unimpaired late-middle aged adults enrolled in the Wisconsin Registry for Alzheimer’s Prevention (WRAP) [42]. Results of the study showed that A + T + adults demonstrated a rate of cognitive decline three times faster than those with a single or no abnormal AD biomarkers over 8 years [42]. Other longitudinal studies have similarly shown that cognitively unimpaired older adults classified as A + T + demonstrate a steeper trajectory of cognitive decline compared to individuals with neither, or only one, abnormal AD biomarker, but that even amongst those classified as A + T+, only between 49-54% progressed to MCI and 3.9-20% progressed to all-cause dementia over 3.5 years [44]. This was similarly observed in this study, where A + T + adults with concurrent CVD experienced a sharp trajectory of *ante-mortem* cognitive decline over a period of 10 years, but only ~ 63% (combined total) had received a clinical diagnosis of MCI or dementia at the last visit before death. Findings from clinical practice and research consistently show that the prevalence and rate of cognitive decline in people with abnormal AD biomarkers varies [46, 47]. Our findings extend this work by showing that the prevalence and rate of cognitive decline remains highly variable even amongst those with concurrent Alzheimer’s and CVD pathology, despite this group showing the steepest trajectory of cognitive decline, and being at the highest risk of conversion to MCI or dementia [7, 48]. 

Clinicopathological studies that sought to clarify the contribution of AD and CVD pathologies on cognition have been mixed. One cross-sectional study showed that white matter hyperintensities interacted with Aβ, but not tau, to drive memory impairment in 586 older adults without dementia [49]. Conversely, a recent study of 205 older adults with varying clinical status demonstrated that those with concomitant white matter hyperintensities and AD pathology (A + T+) showed greater rates of cognitive decline compared to A + T + adults without white matter hyperintensities [50]. Older studies have also shown that brain infarction in combination with AD pathology is associated with poorer cognition and a higher prevalence of dementia compared to those without infarcts [51]. The results of this study add to this growing body of evidence and suggests that in the presence of CVD pathology, A + T + individuals demonstrate the fastest rate of cognitive decline in the years preceding death, and this is significantly greater than that observed in individuals with only one AD pathology (A + only or T + only). It has previously been suggested that the presence of Aβ triggers the conversion of tau from a normal to abnormal state, and once abnormal, tau amplifies Aβ toxicity, which in turn, accelerates tau spreading, neurodegeneration and cognitive decline, resulting in a neurotoxic ‘feedback loop’ [52–54]. It is therefore possible that in the current sample of patients who underwent autopsy, a higher burden of CVD, in addition to abnormal AD pathology, synergistically promoted cognitive decline, which in turn resulted in greater rates of dementia classification prior to death. This accords with a recent in vivo study consisting of 1229 participants from the Swedish BioFINDER-2 Study, where concomitant microbleeds and Aβ pathology were associated with greater baseline tau load and increased tau accumulation, but only in cognitively unimpaired individuals [55]. 

There are several limitations of this study. Firstly, while we showed that the frequency of recent and/or active TIA and stroke was comparable across groups both at study entry and at the last visit before death, an important limitation of this study is a lack of information on the timing and thus the chronicity of vascular events. In addition, information about prevalent TIA and stroke was not available for all participants, and despite accounting for their effects over time in our longitudinal modelling, it is plausible that all effects were not fully accounted for. As a result, it remains unclear whether symptomatic CVD accelerated cognitive decline before death, particularly in the A + T + group, who may be especially vulnerable to additional brain insults, in line with propositions set out by cognitive reserve theory [56]. Next, the A − T + group was the oldest among all groups at the last visit before death (mean age = 89.8 years). Although age was included as a covariate in all statistical models, observed effects may partially reflect age differences across the autopsy groups, which statistical adjustments could not entirely account for. Older age is associated with an increase in dementia risk and cognitive decline attributable to the accumulation of multiple pathologies beyond those measured in this study, including neocortical Lewy bodies, TAR DNA-binding protein 43 (TDP-43) aggregations, and hippocampal sclerosis [45]. As a result, these unmeasured pathologies may have also influenced the rate of cognitive trajectories in this group. Thirdly, a comprehensive range of CVD pathologies were distilled into a composite using a previously validated approach [33]. However, this inhibited the ability to disentangle the individual contributions of each specific marker to cognitive outcomes. Future studies will be needed to investigate the distinct roles and interactions of individual CVD markers with AD pathologies to better understand their effects on cognitive trajectories and to inform intervention strategies targeting cardiovascular and cerebrovascular risk factors. Finally, it is possible that selection bias has influenced who is enrolled in and participated in this study, such that individuals who consent to and are brought to autopsy may not be fully representative of the general population and thus the results of this study may be limited in their generalizability to other cohorts with alternate demographic, clinical, and pathological characteristics [57]. 

These limitations notwithstanding, the results from this study indicate that in individuals with concomitant CVD, the presence of abnormal Aβ and tau at autopsy is associated with the greatest rate of *ante-mortem* cognitive decline, particularly pronounced on the PACC, that is up to ten times faster than A − T− adults. Despite this, the prevalence of clinical progression to MCI and dementia remains highly variable, even amongst those classified as most at risk in this study (i.e., A + T + CVD+), possibly reflecting the influence of other resilience factors (e.g., genetics, lifestyle differences, cognitive reserve) in some individuals compared to others. Whilst it was beyond the scope of this study, it will be important for future studies to further examine these resilience factors in A + T + CVD + individuals who did not progress to dementia prior to death, as this may inform prevention efforts in those at-risk of, but who have not yet developed, dementia. The results of this study also have important implications for clinicopathological models of AD as they challenge recent suppositions that only abnormal tau, and not amyloid, is necessary for cognitive decline.

## Electronic supplementary material

Below is the link to the electronic supplementary material.

Supplementary Material 1

## Data Availability

NACC data is available for use upon written request (https://naccdata.org/requesting-data/data-request-process). Authors who access the data are required to sign and comply with a data use agreement.
