# Supplementary material for: Ante-mortem cognitive trajectories associated with Aβ and tau biomarker profiles in older adults with cerebrovascular disease: a longitudinal cohort study
Source: Alzheimers Res Ther. 2025 Jul 18;17:165. doi: 10.1186/s13195-025-01776-w (PMC12273324; doi:10.1186/s13195-025-01776-w)
Supplement: Supplementary file 1 — Supplementary Material 1 [file 13195_2025_1776_MOESM1_ESM.docx]

**Additional File 1**

**Additional Methods**

We excluded those participants with severe neurological or neuropsychiatric conditions (excluding MCI or dementia) with potential to influence cognitive outcomes, including a clinical diagnosis of traumatic brain injury, normal-pressure hydrocephalus, epilepsy, psychiatric disorders (major depressive disorder, bipolar disorder, schizophrenia or other psychosis, anxiety disorders, post-traumatic stress disorder and any other psychiatric disease), as well as those with cognitive impairment due to alcohol and/or substance abuse, systemic disease or medical illness, or medication use.

**Additional Results**

**Additional Table 1.** A/T group pairwise comparisons across measures of Aβ (CERAD semi-quantitative scores) and tau (BRAAK staging scores) at autopsy.

| **Contrast** | **CERAD Score** | **t-ratio** | ***p*** |
| --- | --- | --- | --- |
| A−T− vs. A+T+ | **0.43 vs. 2.67** | **-51.0** | **<.000** |
| A+T− vs. A+T+ | **2.51 vs. 2.67** | **-3.3** | **.006** |
| A−T− vs. A−T+ | 0.43 vs. 0.54 | -2.2 | .135 |
| **Contrast** | **BRAAK Score** | **t-ratio** | ***p*** |
| A−T− vs. A+T+ | **1.49 vs. 4.38** | **-37.5** | **<.000** |
| A+T− vs. A+T+ | **1.64 vs. 4.38** | **-31.6** | **<.000** |
| A−T− vs. A−T+ | **1.49 vs. 3.77** | **-2.3** | **<.000** |

Abbreviations: CERAD = Consortium to Establish a Registry for Alzheimer’s Disease.

**Additional Table 2.** A/T group pairwise comparisons in cross-sectional PACC performance.

| **Hypothesis 1** | | | | | |
| --- | --- | --- | --- | --- | --- |
| **Contrast** | **PACC EMMs** | **t-ratio** | **Cohen’s d** | **Cohen’s d 95% CI** | ***p*** |
| A−T− vs. A+T+ | **0.07 vs. -0.19** | **-2.99** | **.24** | **[0.06, 0.42]** | **.02** |
| A+T− vs. A+T+ | -0.07 vs. -0.19 | -1.30 | -.10 | [-0.30, 0.09] | .56 |
| **Hypothesis 2** | | | | | |
| **Contrast** | **PACC EMMs** | **t-ratio** | **Cohen’s d** | **Cohen’s d 95% CI** | ***p*** |
| A−T+ vs. A−T− | -0.09 vs. 0.07 | -1.61 | .13 | [-0.08, 0.35] | .37 |

Abbreviations: PACC = Preclinical Alzheimer’s Cognitive Composite, EMMs = estimated marginal means, CI = confidence interval.

**Additional Table 3.** A/T group pairwise comparisons in cross-sectional episodic memory performance.

| **Hypothesis 1** | | | | | |
| --- | --- | --- | --- | --- | --- |
| **Contrast** | **EM EMMs** | **t-ratio** | **Cohen’s d** | **Cohen’s d 95% CI** | ***p*** |
| A−T− vs. A+T+ | **0.11 vs. -0.14** | **-2.68** | **.23** | **[0.05, 0.40]** | **.04** |
| A+T− vs. A+T+ | -0.06 vs. -0.14 | -0.73 | .01 | [-0.19, 0.21] | .88 |
| **Hypothesis 2** | | | | | |
| **Contrast** | **EM EMMs** | **t-ratio** | **Cohen’s d** | **Cohen’s d 95% CI** | ***p*** |
| A−T+ vs. A−T− | -0.11 vs. 0.11 | -2.07 | .22 | [0.00, 0.44] | .16 |

Abbreviations: EM = episodic memory, EMMs = estimated marginal means, CI = confidence interval.

**Additional Table 4.** A/T group pairwise comparisons in cross-sectional executive function performance.

| **Hypothesis 1** | | | | | |
| --- | --- | --- | --- | --- | --- |
| **Contrast** | **EF EMMs** | **t-ratio** | **Cohen’s d** | **Cohen’s d 95% CI** | ***p*** |
| A−T− vs. A+T+ | **0.07 vs. -0.10** | **-2.55** | **.23** | **[0.05, 0.40]** | **.05** |
| A+T− vs. A+T+ | -0.11 vs. -0.10 | 0.11 | .01 | [-0.19, 0.21] | .99 |
| **Hypothesis 2** | | | | | |
| **Contrast** | **EF EMMs** | **t-ratio** | **Cohen’s d** | **Cohen’s d 95% CI** | ***p*** |
| A−T+ vs. A−T− | -0.04 vs. 0.07 | -1.43 | .15 | [-0.07, 0.36] | .48 |

Abbreviations: EF = executive function, EMMs = estimated marginal means, CI = confidence interval.
